# Expression of Multiple Artificial MicroRNAs from a Chicken miRNA126-Based Lentiviral Vector

**DOI:** 10.1371/journal.pone.0022437

**Published:** 2011-07-18

**Authors:** Steve C.-Y. Chen, Patrick Stern, Zhuyan Guo, Jianzhu Chen

**Affiliations:** Koch Institute for Integrative Cancer Research and Department of Biology, Massachusetts Institute of Technology, Cambridge, Massachusetts, United States of America; Max-Planck-Institute for Terrestrial Microbiology, Germany

## Abstract

**Background:**

The use of RNAi in both basic and translational research often requires expression of multiple siRNAs from the same vector.

**Methods/Principal Findings:**

We have developed a novel chicken miR126-based artificial miRNA expression system that can express one, two or three miRNAs from a single cassette in a lentiviral vector. We show that each of the miRNAs expressed from the same lentiviral vector is capable of potent inhibition of reporter gene expression in transient transfection and stable integration assays in chicken fibroblast DF-1 cells. Transduction of Vero cells with lentivirus expressing two or three different anti-influenza miRNAs leads to inhibition of influenza virus production. In addition, the chicken miR126-based expression system effectively inhibits reporter gene expression in human, monkey, dog and mouse cells. These results demonstrate that the flanking regions of a single primary miRNA can support processing of three different stem-loops in a single vector.

**Conclusions/Significance:**

This novel design expands the means to express multiple miRNAs from the same vector for potent and effective silencing of target genes and influenza virus.

## Introduction

Since its discovery in 1998, RNA interference (RNAi) has not only become an enabling technology for studying gene function but has also provided a new approach for treating several diseases [1], [2]. Short interfering RNA (siRNA), short hairpin RNA (shRNA) and microRNA (miRNA) can be introduced into cells or organisms by direct delivery of synthetic oligonucleotides [3]. shRNA and miRNA can also be introduced into cells or organisms by expression vectors. To date, the most widely used vectors are retrovirus-based because of their transduction efficiency and stable and long-term expression of shRNA or miRNA following integration into the host cell genome [4], [5]. Among retroviral vectors, lentiviral vectors have been studied extensively and shown to be effective in expressing shRNAs and miRNAs in animals [6], [7].

Application of RNAi in both basic and translational research often requires expression of multiple siRNAs from the same lentiviral vector. In one of the early designs, ter Brake et al constructed vectors where each of the multiple shRNA expression cassette was driven by a U6 polymerase III (Pol III) promoter [8]. Although multiple shRNAs were expressed simultaneously from the same vector, repetition of the promoter sequences led to deletion of the expression cassettes during lentivirus production. To prevent recombination, the authors constructed vectors where shRNAs were driven by different Pol III promoters with no significant sequence homology. Specifically, three human Pol III promoters U6, H1 and 7SK and one human Pol II promoter U1 were used to express four shRNAs specific for HIV [9]. The stably transduced cells expressed four anti-HIV shRNAs and were shown to delay the emergence of resistant viruses in cell cultures.

Another approach to multiple siRNA expression was stimulated by report that a mouse miR30-based shRNA expression cassette can be driven by Pol II promoters and provide higher knockdown efficiency than those driven by the Pol III U6 promoter [10]. The combination of Pol II promoters and miRNA-based design offers some significant advantages. First, the use of tissue-specific or inducible Pol II promoters allows more effective control of the timing and the level of miRNA expression [11]. Second, the Pol II promoter supports expression of both miRNA cassettes and reporter genes, such as GFP, from the same transcript, thus allowing easier tracking of miRNA expression. Third, using artificial miRNA cassettes modeled after endogenous miRNAs seems to avoid the induction of cellular immune responses and apoptosis [12], [13], [14].

Different strategies have been investigated to express multiple artificial miRNAs from the same lentiviral vector. Zhou et al reported that two tandem copies of the miR30-based cassette can be expressed in a single transcript driven by a Pol II promoter [15], [16]. Subsequently, Sun et al showed that a single Pol II promoter can drive three artificial miR30 cassettes to express siRNAs all targeting GFP, resulting in further knockdown of the GFP intensity in the cells [17]. A similar miR30-based approach was utilized by Zhu et al to knockdown multiple genes [18]. In addition to miR30-based designs, mouse miR155-based design has also been used to knockdown multiple genes [19]. Some miRNAs are present in the genome as a cluster, such as the miR17–92 cluster with six pre-miRNAs encoded in a ∼1 kb pri-miRNA. To explore the miRNA polycistrons for artificial miRNA expression [20], four different anti-HIV artificial miRNAs under the control of the CMV promoter were expressed simultaneously and shown to inhibit virus production in transduced cells. Despite the progress, application of RNAi technology requires more options to express multiple shRNAs or miRNAs from the same lentiviral vector.

In our development of lentiviral vectors capable of expressing multiple anti-influenza miRNAs, we have developed a novel chicken miR126-based miRNA expression cassette. The miR126-based cassette can express one, two or three miRNAs from a single cassette in the context of a lentiviral vector. We show that each of the miRNAs expressed from the same lentiviral vector is capable of potent inhibition of reporter genes in both transient transfection and stable integration assays. Cells transduced with lentivirus expressing two or three, but not one, anti-influenza miRNAs also inhibit influenza virus production. Although the expression cassette is based on chicken miR126, the resulting lentiviral vectors also effectively inhibit reporter gene expression in human, dog, mouse and monkey cells. These results demonstrate the versatility of the miR126-based miRNA expression cassette for potent and effective silencing of target genes.

## Results

### Expression of NP miRNA from the mouse miR30-based lentiviral vector

**Figure 1:**
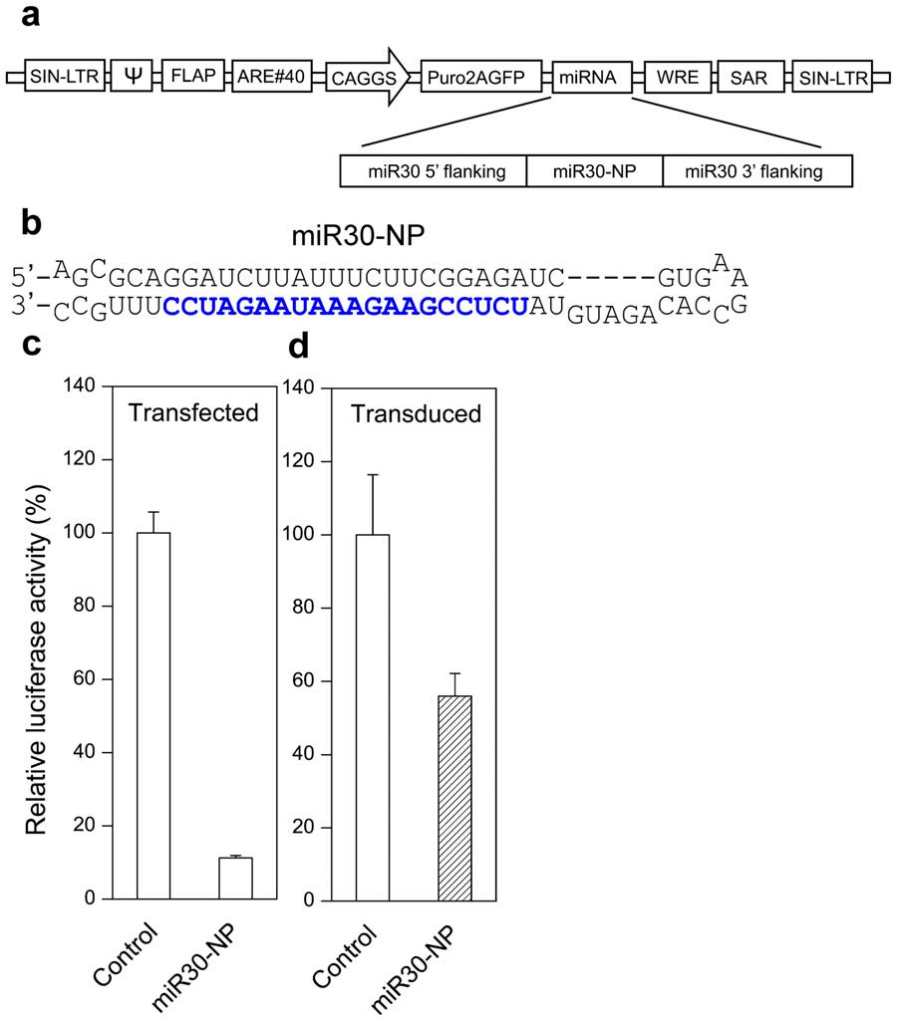
Inhibition of luciferase activity by NP miRNA expressed from a mouse miR30-based lentiviral vector. (**a**) Schematic diagram of the miR30-NP lentiviral vector. The backbone of the vector is pBL2. (**b**) Sequence of miR30-NP hairpin. Blue color letters represent the antisense sequences targeting influenza NP transcript. Flanking and hairpin sequences are miR30. (**c**) Inhibition of luciferase activity by transient transfection of miR30-NP lentiviral vector in DF-1 cells. Psicheck-2 dual luciferase reporter plasmid (50 ng) and miR30-NP lentiviral vector (450 ng) were co-transfected in DF-1 cells. Firefly and Renilla luciferase activities were measured 48 hrs later. Shown are relative Renilla to firefly luciferase activities after normalization to the ratio of control reporter plasmid without target sequence (set as 100%). (**d**) Inhibition of luciferase activity by stably integrated miR30-NP lentiviral vector in DF-1 cells. Cells were infected with lentivirus at an MOI of 0.1 then selected with puromycin and further sorted for GFP-positive cells (>95%). The transduced cells were then transfected with psicheck-2 dual luciferase reporter plasmid (50 ng) and a control plasmid pUC18 (450 ng). Luciferase activity was measured and normalized as in (**c**). All bar graphs represent means ± standard deviations (SD) of three independent experiments.

The mouse miR30-based miRNA expression cassette has been widely used to express artificial miRNA in lentiviral vectors [21]. In the pLB2 vector (Figure 1a), the miRNA cassette is under the control of the RNA polymerase II promoter (CMV-enhancer chicken beta-actin promoter-CAGGS) and transcribed as part of the dual selection marker Puro-2A-GFP transcript. To express anti-influenza artificial miRNA, we replaced the mature miR30 sequences in pLB2 with sequences that target nucleoprotein (NP) of influenza virus (Figure 1b). Anti-influenza RNAi activity was evaluated by dual luciferase assays following either transient transfection or stable integration of the lentiviral vector in chicken embryonic fibroblast DF-1 cells. In the transient transfection assay, the miR30-NP lentiviral vector and psicheck-2 dual luciferase reporter plasmid, in which the NP target sequence was cloned into the 3′ UTR of the synthetic Renilla luciferase gene, were co-transfected into DF-1 cells. The firefly and Renilla luciferase activities were measured 48 hrs later and normalized to the ratio of reporter plasmid without target sequence (set as 100%). As shown in Figure 1c, transient expression of miR30-NP inhibited Renilla luciferase activity by ∼85%. In the stable integration assay, we produced lentivirus and infected DF-1 cells at low multiplicity of infection (MOI = 0.1). The percentage of GFP positive cells was <10% two days after infection (data not shown), suggesting single proviral integration in the majority of the cells. Transduced cells were selected with puromycin and further purified by sorting for GFP positive cells (>95%). Luciferase activity was then measured at 48 hrs following transfection of psicheck-2 reporter plasmid into the GFP-positive cells. After normalization, inhibition of Renilla luciferase activity was about 45% (Figure 1d). These results suggest that although NP miRNA can be expressed from the mouse miR30-based cassette in DF-1 cells, the level of target gene knockdown is modest following stable integration of the lentiviral vector.

### Expression of the endogenous chicken miRNAs from lentiviral vectors

**Figure 2:**
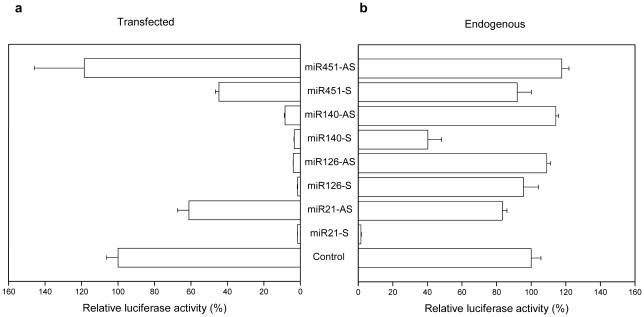
Inhibition of luciferase activity by endogenous and lentiviral expressed chicken miRNAs. (**a**) Inhibition of luciferase activity by lentiviral expressed chicken miRNAs. Endogenous chicken miRNAs plus ∼200 bp flanking sequences on both sides were cloned into pLB2 vector. The corresponding sense and antisense target sequences were cloned into psicheck-2 dual luciferase reporter plasmid. DF-1 cells were transfected with lentiviral vector (450 ng) and the corresponding reporter plasmid (50 ng) and luciferase activities were measured 48 hrs later. Shown are relative Renilla luciferase activities (means ± SD, n = 3) of chicken miRNA expressed from the sense (S) and antisense (AS) strands. (**b**) Inhibition of luciferase activity by endogenously expressed miRNAs. DF-1 cells were co-transfected with 450 ng pUC-18 (as transfection control) and 50 ng psicheck-2 reporter plasmids with endogenous miRNA target sequences and luciferase activities were measured as above. Shown are relative Renilla luciferase activities (means ± SD, n = 3) of sense (S) or antisense (AS) strand of miRNAs.

We reasoned that using chicken miRNA-based expression cassettes in the lentiviral vector may improve knockdown efficiency in transduced chicken DF-1 cells. Based on literature reports and the miRNA database (miRBase), we chose four endogenous chicken miRNAs gga-miR21, gga-miR126, gga-miR140 and gga-miR451 that are expressed in many different tissues of adult chicken and chicken embryo [22]. These four chicken miRNAs plus ∼200 bp flanking sequences on either side of the miRNA stem-loop were amplified by PCR and cloned into the pLB2 lentiviral vector. The chicken miRNA lentiviral vectors and their corresponding sense or antisense psicheck-2 reporter plasmids were co-transfected into DF-1 cells and luciferase activities were assayed 48 hrs later. Expression of both the sense and antisense strands of gga-miR21, gga-miR126 and gga-miR140 led to the inhibition of Renilla luciferase activity (Figure 2a). Expression of the sense but not the antisense strand of gga-miR451 inhibited Renilla luciferase activity, consistent with the report from miRBase [23].

Because gga-miR21, gga-miR126, gga-miR140 and gga-miR451 are generally expressed, we assayed their activity in DF-1 cells by directly transfecting the reporter plasmids into DF-1 cells. As shown in Figure 2b, Renilla luciferase activity was inhibited by ∼98% by the sense strand of gga-miR21, ∼20% by the antisense strand of gga-miR21, and ∼60% by the sense strand of gga-miR140. In contrast, the other endogenous miRNAs did not significantly inhibit the luciferase activity. Comparing the inhibition of luciferase activity by the lentiviral expressed and endogenously expressed miRNAs, these results show that 1) the lentiviral expressed miR126 (both sense and antisense strands) and miR140 antisense strand exhibit potent RNAi activity; 2) the lentiviral expressed miR451 antisense strand does not have any RNAi activity; 3) the observed RNAi activity of lentiviral expressed miR21 (both sense and antisense strands) and miR140 sense strand could be due to endogenous miRNAs. These results suggest that chicken miRNA-based lentiviral vectors could be developed to express anti-influenza miRNAs.

### Expression of NP miRNA via chicken miRNA-based lentiviral vectors

**Figure 3:**
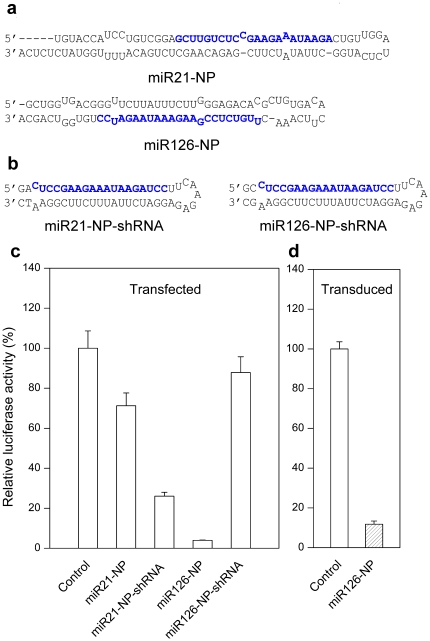
Inhibition of luciferase activity by NP miRNA expressed from chicken miRNA-based lentiviral vectors. (**a**) Structures and sequences of the miR21-NP and miR126-NP. Mature miR21 or miR126 sequences were replaced with anti-influenza NP sequences (blue). (**b**) Structures and sequences of miR21-NP-shRNA and miR126-NP-shRNA. Anti-influenza NP sequences were in blue. (**c**) Inhibition of luciferase activity by NP miRNA expressed from lentiviral vectors from a and b. DF-1 cells were co-transfected with lentiviral vector and the corresponding reporter plasmid, and luciferase activity was measured 48 hrs after transfection. Shown are average relative Renilla luciferase activities (n = 3). (**d**) Inhibition of luciferase activity by stably expressed NP miRNA. DF-1 cells were infected with miR126-NP lentivirus (MOI = 0.1) and were selected with puromycin until GFP-positive cells reached >95%. Cells were then transfected with reporter plasmid and luciferase activity was assayed 48 hrs later. Average Renilla luciferase activity is shown (n = 3).

Based on these results, we selected gga-miR21 and gga-miR126 to construct lentiviral vectors to express NP miRNA. Two different stem-loop designs were tested for miRNA processing. One design mimics the secondary structure of the original miRNA precursor. According to the miRBase, both sense and antisense strands of gga-miR21 and gga-miR126 can produce mature miRNAs. For gga-miR21, the more abundant one is the sense strand and for gga-miR126, the antisense strand. Therefore, we constructed miR21-NP and miR126-NP lentiviral vectors (Figure 3a) where the anti-influenza NP sequences replaced the miR21 sense or miR126 antisense strand, respectively. Because the length of miR21 and miR126 sequences are different, slightly different anti-influenza NP sequences, both containing a 20 nucleotide core sequence of UUGUCUCCGAAGAAAUAAGA, were used to replace them (Figure 3a). The other design replaces the entire pre-miRNA stem-loop with one that is commonly used to express shRNA driven by Pol III promoters (miR21-NP-shRNA and miR126-NP-shRNA) (Figure 3b) [24].

To examine the RNAi activity of these different designs, we co-transfected the lentiviral vectors and NP psicheck-2 reporter plasmid into DF-1 cells and assayed luciferase activity 48 hrs later. The results showed that miR126-NP inhibited luciferase activity by 95% (Figure 3c); miR21-NP-shRNA by 70%; whereas miR21-NP and miR126-NP-shRNA exhibited only minor or no inhibition. The observed differences in targeting efficacy by the different vector designs could be due to differences in miRNA backbones used and/or slightly differences in NP sequences cloned into them. Because miR126-NP was most potent, we chose it for further evaluation. To test the RNAi activity of miR126-NP in stably integrated DF-1 cells, we transduced DF-1 cells with the miR126-NP lentivirus at an MOI of 0.1. Transduced cells (GFP positive) were transfected with the luciferase reporter plasmid and luciferase activity was measured 48 hrs later. Renilla luciferase activity was inhibited by 85% (Figure 3d).

### Development of lentiviral vectors expressing multiple anti-influenza miRNAs

**Figure 4:**
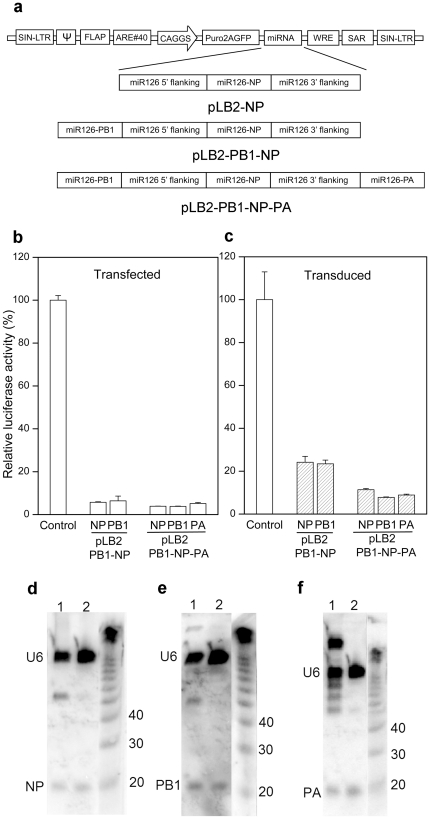
Inhibition of luciferase activity by multiple anti-influenza miRNAs expressed from chicken miR126-based lentiviral vectors. (**a**) Schematic diagram of lentiviral vectors expressing one, two or three anti-influenza miRNAs. pLB2, lentiviral vector backbone; pLB2-NP, pLB2-PB1-NP, and pLB2-PB1-NP-PA, lentiviral vectors expressing NP miRNA, NP and PB1 miRNAs, and NP, PB1 and PA miRNAs, respectively. (**b**) Inhibition of luciferase activity by NP, PB1 or PA miRNAs in transient transfection assays. DF-1 cells were co-transfected with lentiviral vectors (450 ng) and corresponding psicheck-2 reporter plasmids (50 ng). Luciferase activity was measured 48 hrs later. Shown are relative Renilla luciferase activities (means ± SD, n = 3). (**c**) Inhibition of luciferase activity by NP, PB1 or PA miRNAs in stably transduced DF-1. DF-1 cells were transduced with pLB2-PB1-NP or pLB2-PB1-NP-PA lentivirus at an MOI of 0.1. Transduced cells (>95% GFP-positive) were transfected with reporter plasmid with NP, PB1 or PA target sequences. Luciferase activity was measured 48 hrs after transfection. Shown are relative Renilla luciferase activities (means ± SD, n = 3). (**d–f**) Detection of NP, PB1 and PA siRNAs by small RNA Northern blotting. Total RNA was isolated from DF-1 cells that were either transiently transfected with pLB2-PB1-NP-PA lentiviral vector or stably transduced with pLB2-PB1-NP-PA lentivirus. 6 µg RNA from transfected cells (lane 1) or 15 µg of RNA from stably transduced cells (lane 2) were fractionated by electrophoresis on a 15% poly-acrylamide gel. Following transfer, the blots were hybridized with NP, PB1 or PA specific probes and imaged. U6 RNAs were also probed as a loading standard. Molecular weight markers, U6 RNA, and NP, PB1 or PA siRNAs are indicated. Note, the processed PB1 miRNA ran slightly slower than NP and PA miRNAs.

Based on the miR126-NP stem-loop design, we constructed miR126-PB1 and miR126-PA, encoding miRNAs targeting influenza polymerase components PB1 and PA, respectively. The miR126-PB1 stem-loop was inserted at the 5′ end of the miR126 flanking sequence in pLB2-NP, producing lentiviral vector pLB2-PB1-NP (Figure 4a). The miR126-PA stem-loop was inserted at the 3′ end of the miR126 flanking sequence in pLB2-PB1-NP, producing lentiviral vector pLB2-PB1-NP-PA. RNAi activity of NP, PB1 and PA in these vectors was compared by transient transfection assays in DF-1 cells using reporter plasmids that harbored only the NP or PB1 or PA target sequence. Luciferase activity was inhibited by 95% regardless whether NP or PB1 or PA miRNA was expressed from lentiviral vectors expressing NP and PB1, or NP, PB1 and PA (Figure 4b). The observed RNAi activities were specific as pLB2-NP inhibited only NP, but not PB1 or PA, reporter activity, and pLB2-PB1-NP inhibited only NP and PB1, but not PA, reporter activity (Figure S1). In DF-1 cells that were stably transduced with the pLB2-PB1-NP or pLB2-PB1-NP-PA lentivirus, NP, PB1 and PA reporter activity was also inhibited by 80–90% (Figure 4c). Consistent with the RNAi activity, we detected the fully processed NP, PB1 and PA siRNAs by small RNA Northern blotting in DF-1 cells that were transiently transfected with pLB2-PB1-NP-PA lentiviral vector or stably transduced with pLB2-PB1-NP-PA lentivirus (Figure 4d–f). These results show that three artificial miRNAs can be expressed from a single cassette of the chicken miR126-based lentiviral vector.

### Inhibition of influenza virus production by stably expressed anti-influenza miRNAs

**Figure 5:**
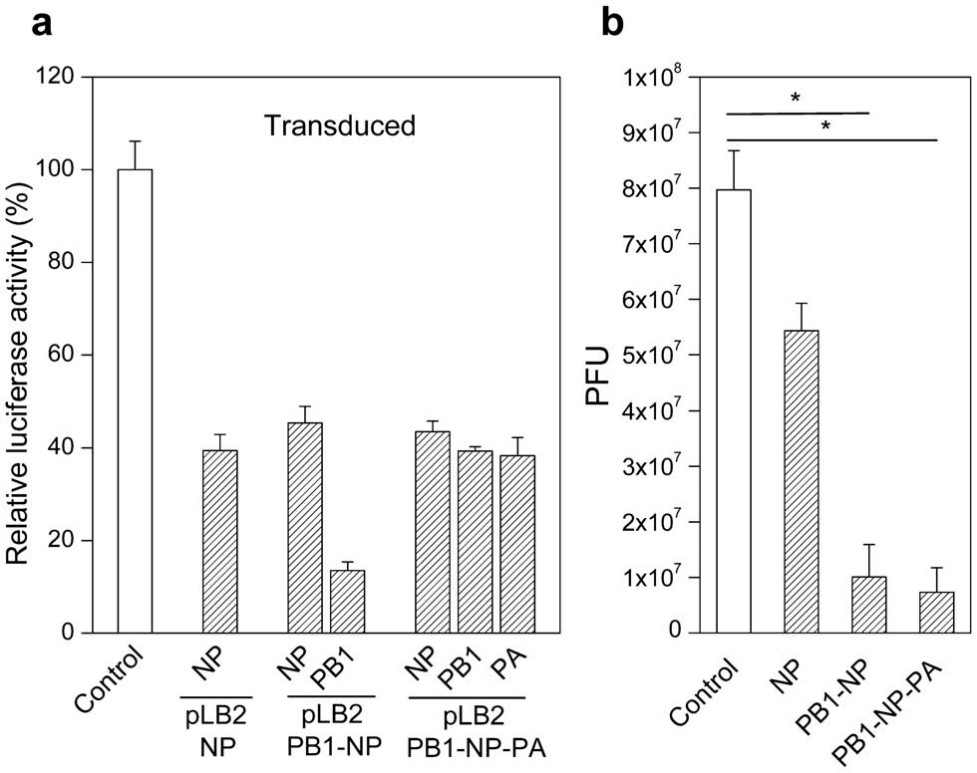
Inhibition of influenza virus production in transduced Vero cells expressing multiple artificial anti-influenza miRNAs. Vero cells were transduced with pLB2-NP, pLB2-PB1-NP, pLB2-PB1-NP-PA, and CPGM (Control) lentiviruses and selected with puromycin to establish transduced cells (>95% GFP-positive). (**a**) The transduced cells were transfected with the indicated reporter plasmids to determine inhibition of luciferase activity. Shown are relative Renilla luciferase activities (means ± SD, n = 3). (**b**) Stably transduced cells were infected with PR8 virus at an MOI of 0.01. 48 hour after infection, the supernatants were collected and assayed for virus titer (PFU) by plaque assay on MDCK cells. Shown are average (means ± SD, n = 3) virus titers per milliliter supernatants. * P<0.001 by two way t-test.

Next, we tested whether the lentivirus-expressed anti-influenza miRNAs are capable of inhibiting influenza virus production. For this purpose, we used Vero cells, from which type I interferon genes had been deleted and are ideal for testing virus inhibition by RNAi. Vero cells were transduced with pLB2-NP, pLB2-PB1-NP, and pLB2-PB1-NP-PA lentiviruses at an MOI of 0.1. As a control, Vero cells were transduced with a CPGM lentivirus that expressed miR30-based miRNA specific for the firefly luciferase transcript. Stably transduced Vero cells (>95% GFP-positive) were tested for inhibition of luciferase activity following transfection of appropriate reporter plasmids (Figure 5a). Inhibition of luciferase activity was significant (by 60–90%) but less than in transduced DF-1 cells, probably due to differences between the two cell types. Transduced Vero cells were infected with the PR8 strain of influenza A virus at an MOI of 0.01 and supernatants were collected 48 hours later for virus titer assay. Transduced Vero cells expressing NP only did not show a significant reduction in viral titer (Figure 5b), whereas transduced Vero cells expressing either NP and PB1, or NP, PB1 and PA showed a reduction in viral titer of approximately 10-fold compared to control CPGM transduced Vero cells.

To exclude the possibility that the anti-viral activity is due to a non-specific effect of flanking sequences that improve processing of miR126-NP, we constructed pLB2-ScrA-NP lentiviral vector where a scrambled sequence replaced PB1, and pLB2-ScrA-NP-ScrB lentiviral vector where two scrambled sequences replaced PB1 and PA (Figure S2a and S2b). Stably transduced Vero cells were examined for luciferase activity following transfection of NP reporter plasmid. All three vectors (pLB2-NP, pLB2-ScrA-NP and pLB2-ScrA-NP-ScrB) inhibited NP reporter activities to the same extent (Figure S2c), suggesting that inclusion of additional flanking sequences does not improve processing of miR126-NP. We further challenged transduced Vero cells with influenza viruses and observed no significant difference in influenza virus inhibition (Figure S2d). Together, these results demonstrate that expression of multiple anti-influenza miRNAs from stably integrated lentiviral vectors is capable of inhibiting influenza virus production.

### The gga-miR126 based lentiviral vector design also works in other cell types

**Figure 6:**
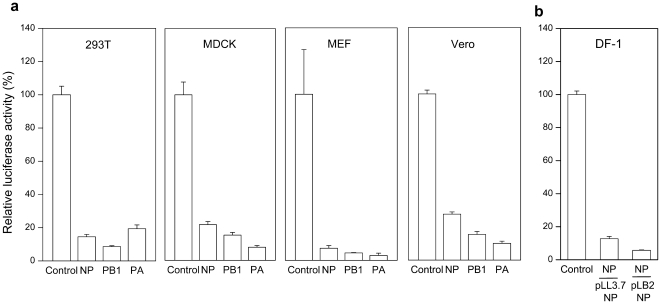
Inhibition of luciferase activity in non-chicken cells by miRNAs expressed from the miR126-based lentiviral vector. (**a**) Inhibition of luciferase activity in 293T, MDCK, MEF and Vero cells. Cells were co-transfected with pLB2-PB1-NP-PA lentiviral vector (450 ng) and the appropriate reporter plasmid (50 ng). Luciferase activity was measured 48 hrs later. Shown are relative Renilla luciferase activities (means ± SD, n = 3). (**b**) Inhibition of luciferase activity by miR126-NP transcribed from the U6 promoter. DF-1 cells were co-transfected with either pLL3.7-NP or pLB2-NP lentiviral vector plus the reporter plasmid. Luciferase activity was assayed 48 hrs later. Shown are relative Renilla luciferase activities (means ± SD, n = 3).

Since RNAi processing machinery is highly conserved among different species, we tested whether the chicken miR126-based lentiviral vector also works in other cell types including human epithelial cell 293T, Madin-Darby Canine Kidney (MDCK) cells, mouse embryonic fibroblast (MEF) cells, and African green monkey kidney (Vero) cells. Luciferase activity was measured in these cell types following transient transfection with the pLB2-PB1-NP-PA lentiviral vector and appropriate reporter plasmids. Luciferase activity was inhibited by NP, PB1 and PA miRNAs by 70 to 95% (Figure 6a), suggesting that the chicken miR126-based lentiviral vector is a general platform for expressing artificial miRNAs. We also tested whether the miR126-NP stem-loop can be properly processed when transcribed from a RNA polymerase III promoter. Thus, miR126-NP was cloned into the pLL3.7 lentiviral vector under the transcriptional control of the U6 promoter. In addition, six thymidines (T) were added to the 3′ end of the hairpin as a termination signal for the Pol III. The resulting lentiviral vector pLL3.7-NP was co-transfected with the reporter plasmid into DF-1 cells and the luciferase activity was inhibited by ∼90% (Figure 6b), similar to the pLB2-NP vector. Thus, the chicken miR126-based stem-loop hairpin can also be transcribed and processed from a Pol III promoter.

## Discussion

In this study, we investigated miRNA-based designs for expressing multiple artificial miRNAs from a single lentiviral vector. By testing different mouse and chicken miRNAs, different hairpins, and different numbers of miRNAs in the same lentiviral vector, we showed that the chicken miR126-based design supports robust expression of artificial miRNAs and efficient knockdown of target genes both in transiently transfected cells and stably transduced cells (Figure 3c,d). To express multiple miRNAs from a single vector, previously described methods rely on inserting multiple miRNA stem-loop cassettes within the 5′ and 3′ flanking sequence of pre-miRNAs. Here, we show that in the miR126-based lentiviral vector, miRNA stem-loop expression cassettes can be placed on both ends of the flanking sequences of pre-miRNA and still maintain efficient processing to produce RNAi activity. Efficient processing of three stem loops interspersed with two flanking sequences suggests that the flanking sequences facilitate stem-loop recognition, perhaps by recruiting factors that enhance stem-loop processing, rather than a context-specific structural role. Furthermore, our results show that the miR126-based design can be transcribed by either RNA Pol II or Pol III and the resulting miRNAs can be processed to exert RNAi activity. In addition, miR126-based design are also efficiently expressed and processed in mammalian cells. Together, these results suggest that the miR126-based design is novel and efficient for expression and processing of artificial miRNAs for RNAi activity.

MicroRNAs have a dynamic range of expression, extending from <1 copy per cell to >10,000 copies per cell. We show that endogenous gga-miR21 is highly active in the DF-1 cells while gga-miR126 is not (Figure 2b). In a reverse correlation, artificial miRNA transcribed from miR126-based design produced potent RNAi activity while that from miR21-based design did not. This result suggests that the high level of endogenous miRNA expression may interfere with the processing of artificial miRNAs that have identical structures. Perhaps design of artificial miRNAs based on a less abundant miRNA would be a better choice, provided that the minor miRNA could be efficiently processed in the cells of interest.

Our results also shed light on miRNA processing in general. Both the secondary structure of the pre-miRNA stem-loop and flanking sequences play important roles in RNAi processing. We compared identical shRNA hairpins cloned into different miRNA contexts. The miR21-NP-shRNA was able to generate functional RNAi but miR126-NP-shRNA was not (Figure 3c). The difference between the two constructs was merely the flanking sequences surrounding the hairpin, suggesting that the flanking sequences play an important role in guiding shRNA processing. It is interesting to speculate how the loop sequence and miRNA flanking sequences may cooperate to regulate miRNA processing.

The anti-influenza siRNA sequences we used here target conserved regions of the influenza genome and have been shown to potently inhibit virus production in cultured cells [25]. In this study, the transduced cells expressing an artificial miRNA against NP alone (the most abundant protein in the virus) did not significantly inhibit virus production. Unlike synthetic siRNA delivery in which cells have significant amounts of siRNA incorporated into RNAi machinery before infection, transduced cells with NP miRNAs alone did not provide enough protection against influenza viruses. However, significant inhibition was observed in transduced cells expressing two or three different anti-influenza miRNAs, suggesting that simultaneous targeting of different genes of influenza provides better protection against influenza infection. The natural reservoirs of influenza viruses are aquatic birds and the viruses are frequently transmitted from wild species to domestic birds. Upon adaptation to an intermediate host such as pig, the new virus can acquire the ability to infect humans and cause severe diseases. Most of the influenza epidemics and pandemics have been traced to avian sources [26]. One way to prevent transmission of new viruses from avian species to human is to introduce anti-influenza siRNAs that target conserved region of influenza virus genes to domestic birds so as to render them resistant to influenza infection [27]. Development of lentiviral vectors that express multiple anti-influenza siRNAs provides the first step towards this goal.

## Materials and Methods

### Vector construction

Maps, sequences and cloning information for the pLB2 lentiviral vector are available online (Addgene http://www.addgene.org/). Endogenous chicken miRNAs with ∼200 bp flanking sequences were PCR cloned into NotI and EcoRI site of the pLB2 lentiviral vector right after puro-2A-GFP. Primers for PCR are listed in Table 1. Sequences encoding miR21-NP, miR21-NP-shRNA, miR126-NP and miR126-NP-shRNA with 5′ and 3′ flanking sequences (Figure S3) were synthesized and cloned into pUC-57 shutter vector by GenScript (New Jersey). The inserts were released with Not I and Pme I digestion and cloned into the pLB2 vector. Following bacterial transformation, 3 to 4 bacterial clones from each vector were picked and plasmid DNA were purified and sequenced. The sequences of the inserts from all the clones were 100% correct. The miR126-PB1 cassette was cloned into Not I and Swa I sites at the 5′ end of flanking sequence of pLB2-NP lentiviral vector. The miR126-PA cassette was cloned into the Pme I site at the 3′ end of the flanking sequences (Figure 4a). Sequences of these anti-influenza artificial miRNA cassettes are listed in Table 2. To construct pLL3.7-NP, the termination signal (TTTTTT) was added to the 3′ end of miR126-NP and one T was added to the 5′ end of miR126-NP in order to reconstitute the U6 promoter. The cassette was cloned into the HpaI and XhoI site in pLL 3.7 vector. To construct psicheck-2 reporter plasmids, about 30 bp NP, PB1 and PA endogenous influenza sequences (Table 3) were cloned into multiple cloning sites of the psicheck-2 dual luciferase reporter plasmid (Promega) following manufacturer's instruction.

**Table 1:** Sequences of PCR primers for endogenous chicken miRNAs cloning.

gga-miR21 Fwd	5′-GCACAGCGGCCGCCAAACACAAGGGAGGC-3′
gga-miR21 Rev	5′-GCGAATTCGATGGAGCTTTAAGAGATGC-3′
gga-miR126 Fwd	5′-GCACAGCGGCCGCGGTGGCTAGAGAAGGACTG-3′
gga-miR126 Rev	5′-GCGAATTCGAGGGAGTTTCTTAGGCTG-3′
gga-miR140 Fwd	5′-GCACAGCGGCCGCGGTGCTGTGTGGCAC-3′
gga-miR140 Rev	5′-GCGAATTCCAAAAATCTAGCTGCATG-3′
gga-miR451 Fwd	5′-CACAGCGGCCGCGGATATCATCATATACTGTAAGTTCAC-3′
gga-miR451 Rev	5′-CGAATTCCTGTGCCATCTCTGATTTTAC-3′

**Table 2:** Sequences of anti-influenza artificial miRNA cassettes.

miR126-NP	5′–GCTGGTGACGGGTTCTTATTTCTTGGGAGACACGCTGTGACACTTCAAAC**TTGTCTCCGAAGAAATAAGAT** *CC*TGTGGTCAGCA-3′
miR126-PB1	5′–GCTGGTGACGTGTAGATCTGTTCCTCCATTGACGCTGTGACACTTCAAAC**TTCAATGGTGGAACAGATCTTCA**TGTGGTCAGCA-3′
miR126-PA	5′–GCTGGTGACGGCTATTGAGGAGTGGCTGATTACGCTGTGACACTTCAAAC**TTAATCAGGCACTCCTCAATTGC**TGTGGTCAGCA-3′

Sequences targeting influenza virus are in bold.

**Table 3:** Endogenous influenza sequences cloned into the psicheck-2 dual luciferase reporter as the targets for anti-influenza shRNAs and artificial miRNAs.

psicheck-2-NP	5′-GAAGGATCTTATTTCTTCGGAGACAAGC-3′
psicheck-2-PB1	5′-ATGAAGATCTGTTCCACCATTGAAGAGC-3′
psicheck-2-PA	5′-AGCAATTGAGGAGTGCCTGATTAATGATCCCTG-3′

### Cell culture

DF-1, Vero, MDCK cells (obtained from ATCC), primary MEF and 293T [21] were cultured in Dulbecco's modified Eagle's medium supplemented with 10% fetal calf serum, penicillin (100 U/ml) and streptomycin (100 µg/ml).

### Dual Luciferase Assay

Co-transfection experiments were performed in 24-well plates. 1×10^5^ cells were seeded per well in 500 µl medium. 24 hr later, 50 ng psicheck-2 reporter plasmid and 450 ng lentiviral vector or pUC-18 were co-transfected with 1.5 µl *Trans*IT®-LT1 Reagent in 50 µl according to manufacturer's instruction (Mirus). 48 hrs later, firefly and Renilla luciferase activities were analyzed with the Dual-Luciferase reporter assay system (Promega).

### Small RNA Northern Blot

Total cellular RNAs were extracted from transduced cells or transient transfected cells with the mirVana miRNA isolation kit (Ambion) according to the manufacturer's protocol. For northern blot analysis, RNAs were separated on a 15% polyacrylamide denaturing gel, electro-transferred to Hybond-N membrane (Amersham Bioscience, Piscataway, NJ) and crosslinked to the membrane using UV light at a wavelength of 254 nm (1200 µJ×100). The membrane was probed with γ-^32^P-labled LNA oligonucleotide probes (LNA position underlined) NP: 5′- GGATCTTATTTCTTCGGAGACAA-3′, PB1: 5′-TGAAGATCTGTTCCACCATTGAA-3′ PA: 5′-GCAATTGAGGAGTGCCTGATTAA-3′. Signals were detected using a phosphorimager (Molecular Dynamics).

### Lentivirus production and transduction

293T cells were cultured to 80% confluency in 175 cm^2^ culture flask. Lentiviruses were produced by co-transfection of lentiviral vector plasmid (16 µg) and packing plasmids Δ8.9 (8 µg) and VSVg (8 µg) with 96 µl TransIT®-LT1 and 3.2 ml OptiMEM (Gibco BRL) into 293T cells. On the second day, medium was replaced with fresh medium. On the third day, supernatant was collected and cellular debris was removed by low-speed centrifugation. The supernatant was filtered through a 0.45 µm low-protein binding membrane (Pall Life Science). To concentrate the virus, supernatant was ultra-centrifuged at 25,000 rpm for 90 minutes. Supernatant was removed and the virus was resuspended in 100 µl medium overnight. The aliquots of virus solution were stored at −80°C. Note, compared to the viral titer with single miRNA cassette, the viral titers dropped 2–4 fold when lentiviral vector had with two and three miRNA cassettes (Figure S4).

5×10^5^ DF-1 or Vero cells were seeded in 6-well plates 24 hour before transduction. Concentrated lentiviruses were add to the medium with 8 µg/ml polybrene and spun at 2500 rpm at 32°C for 90 mins. Two days after lentivirus infection, 5–10 µg/ml puromycin was add to the medium. About 10 to 14 days post-infection, cells were analyzed for GFP by flow cytometry. If the percentage of GFP-positive cells was below 90% after puromycin selection, cells were further enriched by cell sorting to reach >95% GFP^+^ cells.

### Influenza virus infection and titration

5×10^5^ Vero cells were seeded in a 6-well plate. 24 hours later, cells were infected with PR8 virus at an MOI of 0.01 at room temperature for one hour. After the viral solution was removed, 3 ml of DMEM medium containing 0.3% BSA, pen/strep and 4 µg/ml trypsin was added to each well. To determine viral titer by plaque forming unit (PFU) assay, MDCK cells were seeded at 0.5×10^6^ cells/well in 6 well plates in DMEM (10% FBS, 100 U/mL pen/strep, 2 mM glucose) and allowed to grow to single-layer confluence overnight. Media was aspirated from the wells, and 200 µL of virus-containing samples serially diluted 10-fold in PBS were added onto cells. After one hour incubation with periodic shaking, cells were covered with 2 mL of semi-solid 2% agar/media solution to restrict viral particle spread to cell-to-cell contacts. Plaques were counted after 3 days. Assay was performed in triplicates.

## Supporting Information

Figure S1 **Lentiviral vector-mediated inhibition of reporter gene expression is sequence specific.** DF-1 cells were co-transfected with either pLB2-NP (**a**) or pLB2-PB1-NP (**b**) lentiviral vectors (450 ng) and NP, PB1 or PA psicheck-2 reporter plasmids (50 ng) and luciferase activity was measured 48 hrs later. Shown are relative Renilla luciferase activities (means ± SD, n = 3).
(TIF)

Figure S2 **Flanking sequences do not improve processing of miR126-NP.** (**a**) Schematic diagram of lentiviral vectors: backbone, pLB2-NP, pLB2-ScrA-NP, and pLB2-ScrA-NP-ScrB. miR126-PB and miR126-PA cassettes were replaced with miR126-ScrA and miR126-ScrB (**b**), respectively. The scrambled sequences A and B (in blue) used here do not target influenza genome. (**c**) Vero cells were transduced with pLB2-NP, pLB2-ScrA-NP and pLB2-ScrA-NP-ScrB and sorted for GFP-positive cells (>95%). The transduced cells were transfected with the NP reporter plasmid and luciferase activity was measured 48 hrs later. Shown are relative Renilla luciferase activities (means ± SD, n = 3). (**d**) Stably transduced Vero cells were infected with PR8 virus at MOI of 0.01. 48 hrs after infection, the supernatants were collected and assayed for virus titer by plaque assay on MDCK cells. These results demonstrate that inclusion of flanking sequences does not enhance anti-NP activity by improving processing of miR126-NP.
(TIF)

Figure S3 **Complete sequences of miR21-NP, miR126-NP, miR-NP-shRNA and miR126-NP-shRNA.** NP miRNA and NP shRNA sequences are in blue. Not I and Pme I restriction enzyme sites are underlined.
(PDF)

Figure S4 **Comparison of viral titer between lentiviral vectors with single, double and triple miRNA cassettes.** 293T cells were infected with lentiviruses made from lentiviral vectors with single, double and triple miRNA cassettes. The viral titer dropped 3.8 folds when a second miRNA cassette was added into the pLB2 lentiviral vector and dropped another 2 folds when a third miRNA cassette was added. pLB2-NP (1.2×10^8^ TU/mL), pLB2-PB1-NP (3.2×10^7^ TU/mL) and pLB2-PB1-NP-PA (1.6×10^7^ TU/mL). Shown are transduction unit/mL (means ± SD, n = 5).
(TIF)
