# Supplementary figures and images for: Expression of Multiple Artificial MicroRNAs from a Chicken miRNA126-Based Lentiviral Vector
Source: PLoS One. 2011 Jul 18;6(7):e22437. doi: 10.1371/journal.pone.0022437 (PMC3138786; doi:10.1371/journal.pone.0022437)

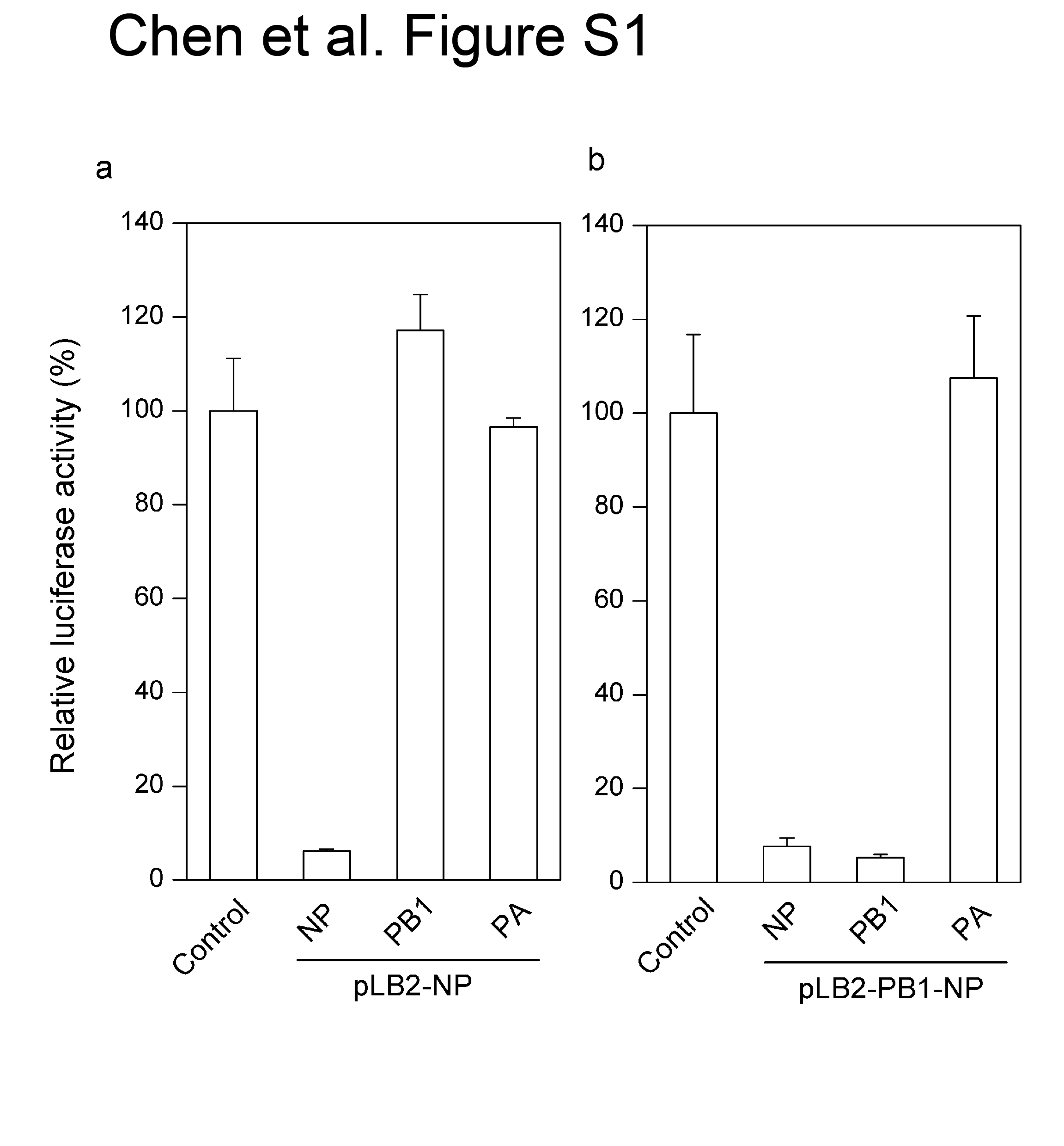

Supplement: Figure S1 — Lentiviral vector-mediated inhibition of reporter gene expression is sequence specific. DF-1 cells were co-transfected with either pLB2-NP (a) or pLB2-PB1-NP (b) lentiviral vectors (450 ng) and NP, PB1 or PA psicheck-2 reporter plasmids (50 ng) and luciferase activity was measured 48 hrs later. Shown are relative Renilla luciferase activities (means ± SD, n = 3). (TIF) [file pone.0022437.s001.tif]

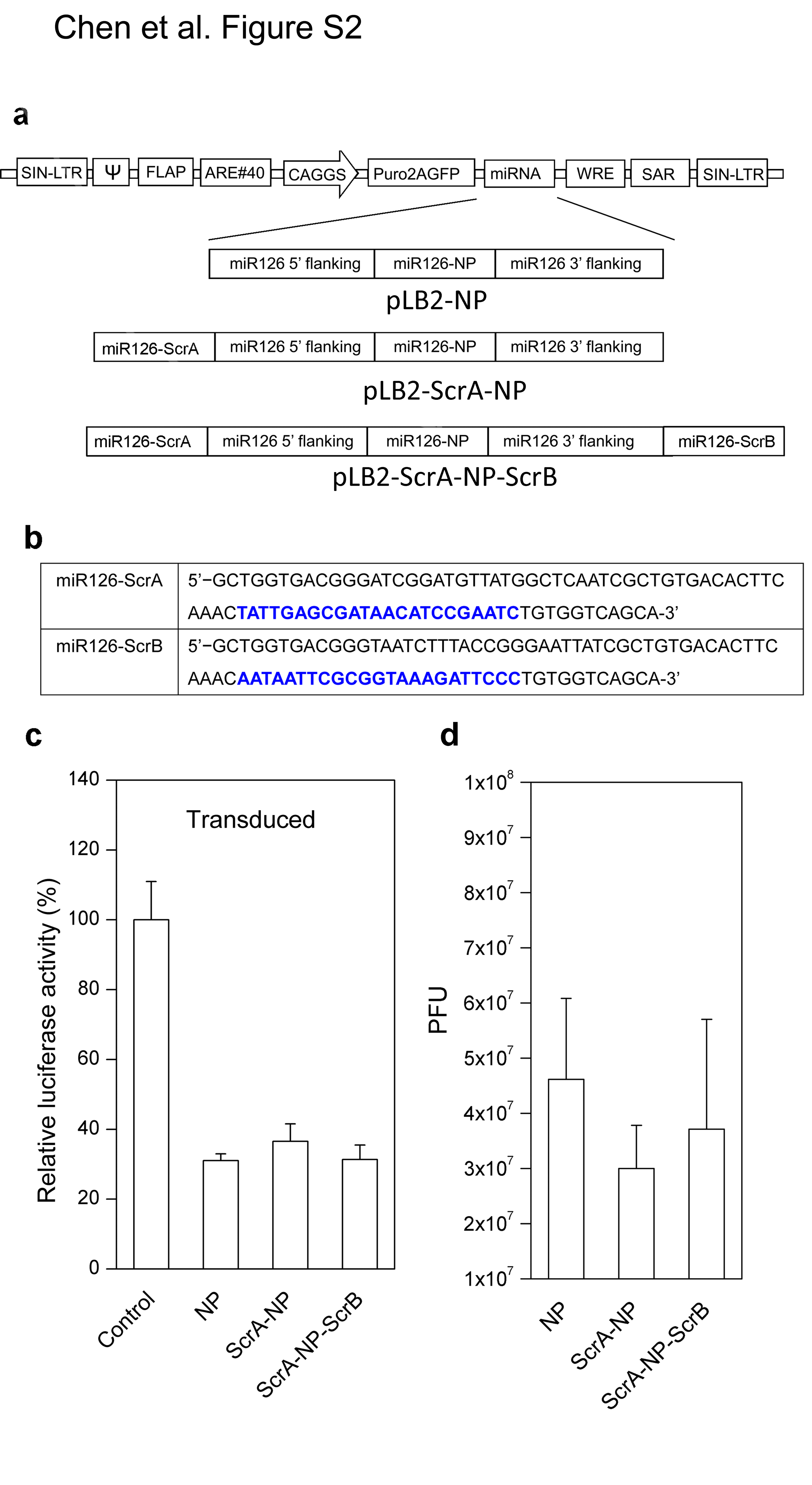

Supplement: Figure S2 — Flanking sequences do not improve processing of miR126-NP. (a) Schematic diagram of lentiviral vectors: backbone, pLB2-NP, pLB2-ScrA-NP, and pLB2-ScrA-NP-ScrB. miR126-PB and miR126-PA cassettes were replaced with miR126-ScrA and miR126-ScrB (b), respectively. The scrambled sequences A and B (in blue) used here do not target influenza genome. (c) Vero cells were transduced with pLB2-NP, pLB2-ScrA-NP and pLB2-ScrA-NP-ScrB and sorted for GFP-positive cells (>95%). The transduced cells were transfected with the NP reporter plasmid and luciferase activity was measured 48 hrs later. Shown are relative Renilla luciferase activities (means ± SD, n = 3). (d) Stably transduced Vero cells were infected with PR8 virus at MOI of 0.01. 48 hrs after infection, the supernatants were collected and assayed for virus titer by plaque assay on MDCK cells. These results demonstrate that inclusion of flanking sequences does not enhance anti-NP activity by improving processing of miR126-NP. (TIF) [file pone.0022437.s002.tif]

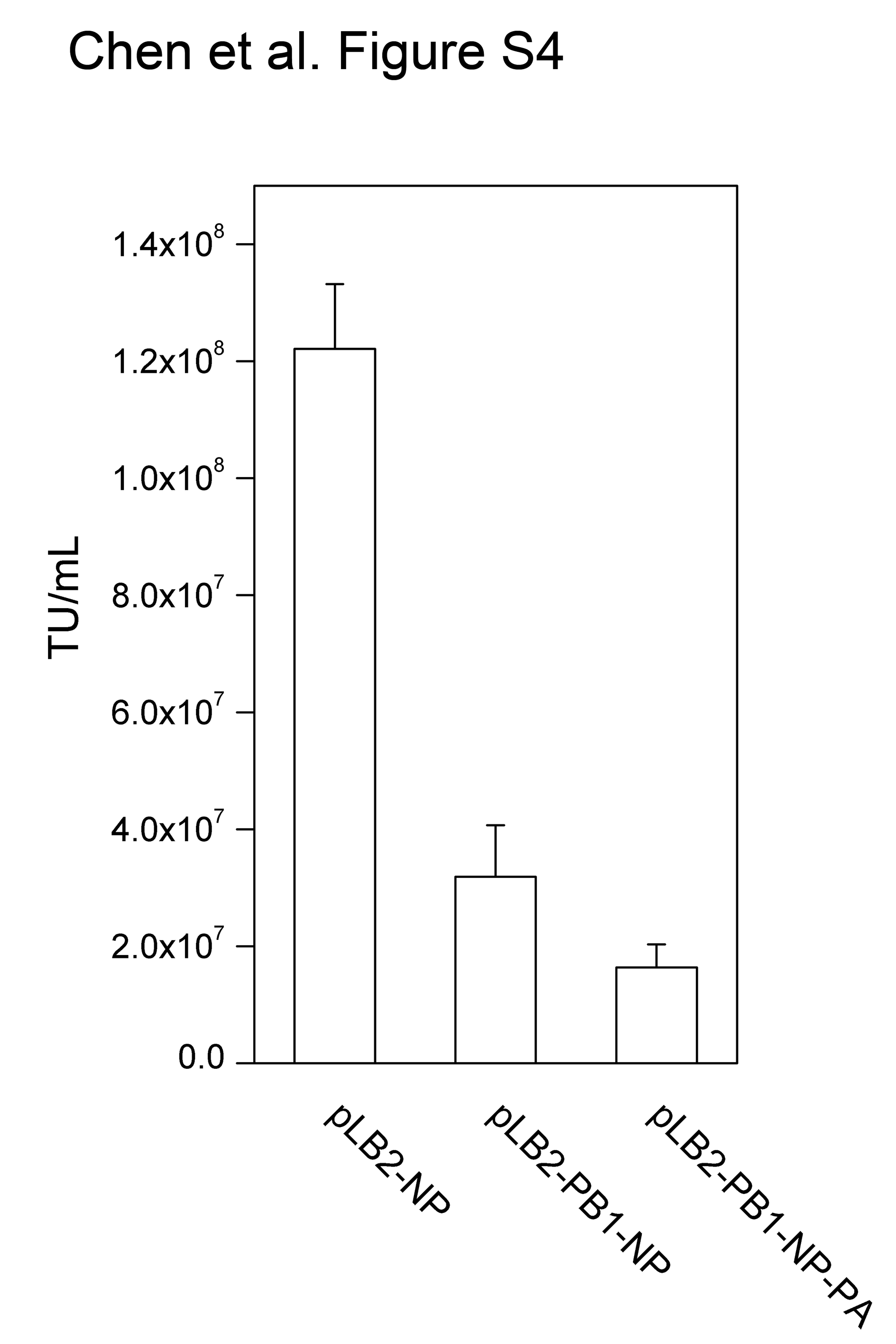

Supplement: Figure S4 — Comparison of viral titer between lentiviral vectors with single, double and triple miRNA cassettes. 293T cells were infected with lentiviruses made from lentiviral vectors with single, double and triple miRNA cassettes. The viral titer dropped 3.8 folds when a second miRNA cassette was added into the pLB2 lentiviral vector and dropped another 2 folds when a third miRNA cassette was added. pLB2-NP (1.2×10^8^ TU/mL), pLB2-PB1-NP (3.2×10^7^ TU/mL) and pLB2-PB1-NP-PA (1.6×10^7^ TU/mL). Shown are transduction unit/mL (means ± SD, n = 5). (TIF) [file pone.0022437.s004.tif]
