# Supplementary material for: Expression of Multiple Artificial MicroRNAs from a Chicken miRNA126-Based Lentiviral Vector
Source: PLoS One. 2011 Jul 18;6(7):e22437. doi: 10.1371/journal.pone.0022437 (PMC3138786; doi:10.1371/journal.pone.0022437)
Supplement: Figure S3 — Complete sequences of miR21-NP, miR126-NP, miR-NP-shRNA and miR126-NP-shRNA. NP miRNA and NP shRNA sequences are in blue. Not I and Pme I restriction enzyme sites are underlined. (PDF) [file pone.0022437.s003.pdf]

Figure S3

miR21-NP (418bp)

GCGGCCGCGCACACAAACACAAGGGAGGCTTGATTTTGCTCACGCTTGCATTGTATTTATCC
CTGCATTAGGTTAGGGCAGATCATGAGCAAGGTGTGAGAATGAGGATTTTGTTCAATCCTGC
CTGAATGTCCTCCTGTGTTGCCAGCCGTCGTGACATCCCCATGGC**TGTACCATCCTGTCGG**
AGCTTGTCTCCGAAGAAATAAGACTGTTGGATCTCATGGCTTATATCTTCGAGACAAG
CTCTGACATTTTGGTATCTCTCATCTGACCGTTTGCTCACCCATCTCCAGCTGTTTTCATT
GAACATCAGTGGCATATAAATGTTTTTCAGTTTTGATGGAAATGAGTACACCAGCCATGCAG
TGTGGCAAGCTGCATCCGATGCATCTCTTAAAGCTCCATCTCGGGTTTAAAC

miR126-NP (498bp)

GCGGCCGCCAGGGTGGCTAGAGAAGGACTGGCAAGGGCTAGGAGATGGAACAAAACCTG
CCTTGTGCACAAGCCCAGGCCCTGCAGGGGTGATAAAGCCTGGCTGTGGTGTGTGGTGGTC
AGGTCTGTGGCTGTGCTCTCCGTCATCATCGTCATTCTTCCTGTGGGGCACATCCATCCGGA
GCCACAAGGAGCATCAGGAGCT**GCTGGTGACGGGTTCTTATTTCTTGGGAGACACGCT**
GTGACACTTCAAACTTGTCTCCGAAGAAATAAGATCCTGTGGTCAGCACTGGCATCAC
GTGGGCAACGCCTGGGAGAGCCATCTACATGCGTCCCAAACACTGCCTGCTTTTGCCCTGT
GTCTGCAGCAGGGCTCACCATGGGACAGGGAAGACATGAGTGCTAACTCATCTGTTGACA
GCAAAGGCACGAAAGGATATTTTGTCCTTGAAGCATCACCCCACAGCCTAAGAAACTCCCT
CACTTTATTCGTTTAAAC

miR21-NP-shRNA (407bp)

GCGGCCGCGCACACAAACACAAGGGAGGCTTGATTTTGCTCACGCTTGCATTGTATTTATCC
CTGCATTAGGTTAGGGCAGATCATGAGCAAGGTGTGAGAATGAGGATTTTGTTCAATCCTGC
CTGAATGTCCTCCTGTGTTGCCAGCCGTCGTGACATCCCCATGGCTGTACCATCCTGTCG**GA**
CTCCGAAGAAATAAGATCCTTCAAGAGAGGATCTTATTTCTTCGGAATCTGACATTTTG
GTATCTCTCATCTGACCGTTTGCTCACCCATCTCCAGCTGTTTTCATTGAACATCAGTGGCAT
ATAAATGTTTTTCAGTTTTGATGGAAATGAGTACACCAGCCATGCAGTGTGGCAAGCTGCAT
CCGATGCATCTCTTAAAGCTCCATCTCGGGTTTAAAC

miR126-NP-shRNA (486bp)

GCGGCCGCCAGGGTGGCTAGAGAAGGACTGGCAAGGGCTAGGAGATGGAACAAAACCTG
CCTTGTGCACAAGCCCAGGCCCTGCAGGGGTGATAAAGCCTGGCTGTGGTGTGTGGTGGTC
AGGTCTGTGGCTGTGCTCTCCGTCATCATCGTCATTCTTCCTGTGGGGCACATCCATCCGGA
GCCACAAGGAGCATCAGGAGCTGCTGGTGACG**GCCTCCGAAGAAATAAGATCCTTCAAG**
AGAGGATCTTATTTCTTCGGAAGCTGTGGTCAGCACTGGCATCACGTGGGCAACGCCTG
GGAGAGCCATCTACATGCGTCCCAAACACTGCCTGCTTTTGCCCTGTGTCTGCAGCAGGGC
TCACCATGGGACAGGGAAGACATGAGTGCTAACTCATCTGTTGACAGCAAAGGCACGAAA
GGATATTTTGTCCTTGAAGCATCACCCCACAGCCTAAGAAACTCCCTCACTTTATTCGTTTAA
AC
